# Effects of L-Glutamine oral supplementation on prostate of irradiated rats

**DOI:** 10.1590/S1677-5538.IBJU.2015.0187

**Published:** 2016

**Authors:** Flavia C. M. Pinto, Waldemar S. Costa, Pamella C. Silva, Diogo B de Souza, Bianca Gregório, Francisco J. B. Sampaio

**Affiliations:** 1 Núcleo de Cirurgia Experimental do Departamento de Cirurgia da Universidade Federal de Pernambuco, PE, Brasil;; 2 Unidade de Pesquisa Urogenital da Universidade Estadual do Rio de Janeiro, UERJ, RJ, Brasil

**Keywords:** L-glutamine 2-deoxy-scyllo-inosose aminotransferase [Supplementary Concept], Prostate, Rats

## Abstract

**Objectives:**

To investigate the protective effect of L-Glutamine in animals undergone to ventral radiation when the target organ is not the prostate.

**Materials and Methods:**

Wistar rats were divided into groups of 10 animals each: Controls (C), maintained under standard conditions and not exposed to radiation, Radiated group (R) undergone to abdominal radiation only and Radiated plus supplemented by L-glutamine group (R+G). The animals of group R+G were supplemented with L-glutamine at the beginning of the experiment until death in the 22nd day. The ventral prostate was dissected and processed for morphometrical analysis. The epithelial height, collagen density and acinar area were objectively assessed in histological sections.

**Results:**

Epithelial height was significantly reduced in R group in comparison to C group (p= 0.005). However, there was no statistical difference between the C and R+G groups. Collagen surface density in the C and R groups were not statistically different, but a significant difference was observed when comparing groups R+G and R (p= 0.040). The R+G group values did not differ significantly from C group. The acinar prostate area of group R was similar to that of C (p= 0.971), but in R+G it was significantly reduced when compared with the C (p= 0.038) and R (p= 0.001) groups.

**Conclusions:**

Pelvic radiation promotes structural modifications in ventral prostate of rats, which can be reduced by L-Glutamine.

## INTRODUCTION

Ionizing radiation, when used to destroy tumor cells in pelvic organs, always affect the normal cells of the target and surrounding organs leading to important side effects. Despite its negative aspects, pelvic radiotherapy is increasingly used for treatment of bladder and rectum cancer. As the consequence, there is a growing incidence of acute and chronic radiation-related lesions in pelvic organs, including the prostate (1).

The L-Glutamine is considered a non-essential amino acid in homeostatic situations but becomes essential in catabolic circumstances such as trauma and sepsis (2, 3). L-Glutamine is metabolized to glutathione that protects tissues against oxidative damage, and acts as a nitrogen conductor between cells and may be precursor for nucleotides and glucose (4). Supplementation with this amino acid prevents bacterial translocation from the intestinal mucosa, reduces the infection rate, hospitalization time and mortality in critically ill patients (4, 5).

Diestel et al. (6) suggests that the L-Glutamine supplementation assists the colonic wall repair in rat’s after radiation. Radiotherapy toxic effects are extended when L-glutamine levels are low (7). Possibly the L-glutamine deficiency may limit both the protein production in inflammatory response and glutathione synthesis compromising the body antioxidant defenses (8).

The aim of the present work is to investigate the effects of radiation over a nonneoplasic prostate and the protective effect of L-Glutamine in this radiated organ.

## MATERIAL AND METHODS

In the present study thirty adult male Wistar rats (90 days old, 350grams of body weight) were kept in a room with controlled temperature (25±1ºC), artificial dark-light cycle (lights on from 7:00 am to 7:00 pm) and fed standard rat chow and water ad libitum.

The rats were randomly allocated into three groups: Control group (C) was maintained under standard conditions and was not exposed to radiation (n=10). Radiated group (R) undergone pelvic radiation (n=10) at the eighth day of the experiment. Supplemented and radiated group (R+G) undergone pelvic radiation plus L-glutamine supplementation (n=10). This group (R+G) was also exposed to radiation at the eighth day of the experiment and was supplemented with L-glutamine (Resource Glutamine, Novartis, Rio de Janeiro, Brazil) from the beginning of the experiment (day 0) until death. L-Glutamine was administered by gavage at a dose of 0.2g/Kg of body weight diluted in distilled water (6).

The animals of the R and R+G groups received a unique dose of abdominal radiation of 1164cGy. All rats were maintained in a dorsal position inside small plastic cages, avoiding movements during pelvic radiation. A linear accelerator of 06 MeV (model Clinac 2100®–Varian®) liberated the radiation with a speed of 240cGy/min, in a font-skin distance of 100cm, in a 6x4cm field over the lower abdomen. The head, thorax and members were excluded of the radiation field.

During all experiment stages, the animals were observed for signs of toxicity such as lack of appetite, weight loss, piloerection, hyper or hypo activity.

All animals were submitted to euthanasia by an overdose of sodium thiopental on the 22^nd^ day (14 days after radiation exposure).

Prostate was dissected under magnification, and its ventral lobe was fixed in 4% buffered formaldehyde. The specimens were processed for paraffin embedding and sections of 5µm thickness were obtained. Samples were stained by hematoxylin and eosin to study acinar structures and Picrosirius red for collagen analysis.

Micrographs were captured by a digital camera (DP70-Olympus®) coupled to a light microscope (BX51-Olympus®). All analyses were performed on random fields with the software ImageJ® (National Institute of Health, USA).

After calibration, the area of the prostatic acini and epithelium height were measured with “freehand selections” and “straight line selections” tools respectively.

For collagen analysis, a 100 points grid was superimposed over the images, and the point counting method (9, 10) was used to objectively determinate collagen surface density, expressed as percentage.

For parametric values, analysis of variance (ANOVA) followed by Student t test were used. For nonparametric data Kruskal-Wallis test, followed by Mann-Whitney test were used. The GraphPad Prism 5.0 software was used for statistical analysis. The significance level for rejecting the null hypothesis was 5% (p≤0.05).

This research was approved by the Institutional Animal Bioethics Committee of the Biological Sciences Center, State University of Rio de Janeiro (protocol number: CEA/224/2008).

## RESULTS

After radiation exposure, all animals presented diarrhea. No other toxicity sign was observed.

**Figure 1:**
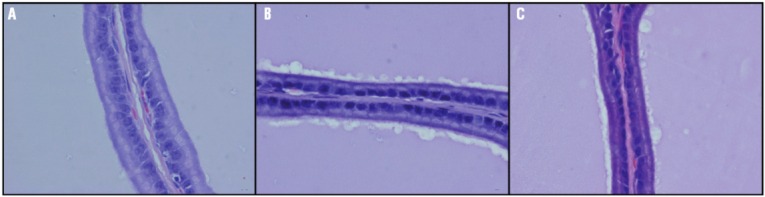
Epithelial height in the ventral prostate. a: Control; b: Irradiated ; c: Irradiated+L-glutamine. HE. X 1000.

Epithelial height was significantly reduced in group R in comparison to group C (p<0.01). In the group R+G the epithelial height was similar to the C group (Table-1, Figure-1).

**Table-1:** Morphometric data of ventral prostate from control, radiated and radiated+L-glutamine supplementation rats.

	Control	Radiated	Radiated+Glutamine
Epithelial height (µm)	18.31±1.9	11.59±0.8^a^	13.14±1.4
Acinar area (µm^2^.10^3^)	78.3±5.2	78.6±2.8	64.1±3.6^a,b^
Collagen density (%)	8.14±0.6	7.00±0.5	9.13±0.8b

Values are presented as mean±SD; **a**: statistically different from Control group; **b**: statistically different from Radiated group.

The collagen density between C and R groups showed no statistical difference (p=0.16). Collagen increased significantly in the group R+G when compared with group R (p=0.04). The R+G group values did not differ significantly from C group (p=0.37) (Table-1).

The prostate acinar area of group R was similar to that of C group (p=0.97). The R+G group had a statistical decrease when compared with C group (p=0.03) and R groups (p<0.01) (Table-1).

According to data, the effect of radiation on the prostate of the rat affected the height of the epithelium significantly. In radiated rats and after supplementation with L-glutamine, it was observed a significant increase in the amount of collagen and a significant decrease in the size of acini (Table-1, Figure-1).

## DISCUSSION

The pelvic radiation is well-recognized to produce major side effects contributing to morbidity of oncologic patients. Most of these effects occur in consequence of radiation to organs without cancer. This can begin immediately after the tissue exposition but the histological modification may take some weeks to occur (1, 11, 12).

In the present study, the tissue modifications after two weeks from radiation were evaluated. This period was determined taking into account that rat’s tissue metabolism is faster when compared with humans. Despite the anatomical differences between human and rodents prostate, there are many similarities that allow the use of the latter as an experimental model, especially in regard to the acinar epithelium (13). Also, the choice of the ventral lobe to be analyzed comes from the statement that this tissue is the most similar to the human prostate (14, 15).

One of the consequences of the radiation-matter interaction on cell’s structures is the production of reactive oxygen species and oxidative damage (16). The immediate radiation effects can be easily observed in tissues with great proliferative capability, such as the epithelium, leading to vascular injuries, hypoxia, and cell death (11).

Radiation promoted a significant change in the prostate acinar epithelium height, decreasing it in approximately 36%, when compared to control animals. This modification corroborates with what was previously pointed out by Stone et al. (11).

Studies in rats showed that L-Glutamine aids in colonic wall healing after radiation (17, 18). However, there is a lack of information regarding L-glutamine in preserving and maintaining the integrity of the prostate after pelvic radiotherapy. The present study establishes through quantitative methods the effects of oral supplementation with L-glutamine for protecting the prostate from radiation.

A study concerning morphometric evaluation of ventral prostate cells of rats growth in primary cultures, determined that L-Glutamine supplemented cultures had a faster cellular growth (19). Actually, L-Glutamine acts on epithelial cells providing an adequate environment for its development. In the present study L-Glutamine was effective in restoring normal epithelium after pelvic radiation.

No significant change was observed on the total area of the acini after radiation. However, in the group supplemented with L-glutamine a 18% reduction in the size of acini was observed. This decrease could be explained by protein synthesis and muscle tissue development stimulated by L-glutamine (20). When muscle matrix density rises, the parenchyma reorganizes and, as a consequence, the acinar size reduces.

L-Glutamine supplementation is involved in extracellular matrix remodeling, influencing the rising collagen synthesis from fibroblasts, myofibroblasts and muscle cells. These cells, when activated act as collagen primary producers and others extracellular matrix components (6). The data presented show that L-Glutamine has a protective effect over prostate extracellular matrix, maintaining normal collagen levels.

One limitation of the present work is the short-term analysis after radiation treatment. It is possible that an analysis after longer period from pelvic radiation could show a more severe change than those observed in this work.

## CONCLUSIONS

Pelvic radiation promotes structural modifications on ventral prostate of rats. These modifications can be reduced by oral supplementation with L-Glutamine.
